# A new rapid diagnostic system with ambient mass spectrometry and machine learning for colorectal liver metastasis

**DOI:** 10.1186/s12885-021-08001-5

**Published:** 2021-03-10

**Authors:** Sho Kiritani, Kentaro Yoshimura, Junichi Arita, Takashi Kokudo, Hiroyuki Hakoda, Meguri Tanimoto, Takeaki Ishizawa, Nobuhisa Akamatsu, Junichi Kaneko, Sen Takeda, Kiyoshi Hasegawa

**Affiliations:** 1 Hepato-Biliary-Pancreatic Surgery Division, Department of Surgery, Graduate School of Medicine, University of Tokyo, 7-3-1 Hongo, Bunkyo-ku, Tokyo, 113-8655 Japan; 2 Department of Anatomy and Cell Biology, Interdisciplinary Graduate School of Medicine and Engineering, University of Yamanashi, Yamanashi, Japan

**Keywords:** Colorectal cancer, Liver metastasis, Rapid diagnosis, Mass spectrometry, Machine learning

## Abstract

**Background:**

Probe electrospray ionization-mass spectrometry (PESI-MS) can rapidly visualize mass spectra of small, surgically obtained tissue samples, and is a promising novel diagnostic tool when combined with machine learning which discriminates malignant spectrum patterns from others. The present study was performed to evaluate the utility of this device for rapid diagnosis of colorectal liver metastasis (CRLM).

**Methods:**

A prospectively planned study using retrospectively obtained tissues was performed. In total, 103 CRLM samples and 80 non-cancer liver tissues cut from surgically extracted specimens were analyzed using PESI-MS. Mass spectra obtained by PESI-MS were classified into cancer or non-cancer groups by using logistic regression, a kind of machine learning. Next, to identify the exact molecules responsible for the difference between CRLM and non-cancerous tissues, we performed liquid chromatography-electrospray ionization-MS (LC-ESI-MS), which visualizes sample molecular composition in more detail.

**Results:**

This diagnostic system distinguished CRLM from non-cancer liver parenchyma with an accuracy rate of 99.5%. The area under the receiver operating characteristic curve reached 0.9999. LC-ESI-MS analysis showed higher ion intensities of phosphatidylcholine and phosphatidylethanolamine in CRLM than in non-cancer liver parenchyma (*P* < 0.01, respectively). The proportion of phospholipids categorized as monounsaturated fatty acids was higher in CRLM (37.2%) than in non-cancer liver parenchyma (10.7%; *P* < 0.01).

**Conclusion:**

The combination of PESI-MS and machine learning distinguished CRLM from non-cancer tissue with high accuracy. Phospholipids categorized as monounsaturated fatty acids contributed to the difference between CRLM and normal parenchyma and might also be a useful diagnostic biomarker and therapeutic target for CRLM.

**Supplementary Information:**

The online version contains supplementary material available at 10.1186/s12885-021-08001-5.

## Background

Colorectal cancer is the third most common cancer worldwide and is ranked as the second most frequent cause of cancer-associated mortality in industrialized countries. Colorectal liver metastasis (CRLM) is the major cause of mortality in patients with colorectal cancer, affecting approximately 50% of patients [1, 2]. The mainstay of treatment for CRLM is complete surgical resection of all metastatic lesions [3–5]. Recent advancements in radiologic imaging techniques, especially intraoperative ultrasonography, have enabled us to identify additional new hepatic nodules in 14 to 24% of patients [6–8]. However, it is sometimes difficult to correctly diagnose whether such new hepatic nodules are CRLM tumors. In such cases, intraoperative biopsy is one option for diagnosis of hepatic nodules. However, the diagnostic quality of frozen sections is sometimes inferior to that of paraffin-embedded sections [9]. Thus, rapid and accurate diagnostic techniques taking place of frozen section are needed.

We recently developed a new diagnostic system that combines probe electrospray ionization-mass spectrometry (PESI-MS) and machine learning [10]. PESI is the one of ionization methods that requires only a few milligrams of a sample without any complicated pretreatments. PESI-MS is a mass spectrometry using PESI, which enables to rapidly obtain results compared to conventional mass spectrometry [11]. The obtained mass spectra are processed using machine learning algorithms such as logistic regression or support vector machines to discriminate cancer from non-cancer tissues [12]. Previous experiments demonstrated high discriminating power for hepatocellular carcinoma and renal cell carcinoma [10, 11]. PESI-MS and machine learning is a cutting-edge diagnostic tool that can detect the difference in lipid profiles between various cancerous and non-cancerous tissues. While PESI-MS can rapidly visualize mass spectra, it cannot identify individual molecules and their pattern of mass spectra. To identify the molecules responsible for the difference between cancerous and non-cancerous samples, we used liquid chromatography-electrospray ionization-MS (LC-ESI-MS), which provides a more detailed view of sample molecular compositions.

Our present study aims to validate PESI-MS and machine learning for the rapid diagnosis of CRLM.

## Methods

### Patients and sample collection

This is a prospectively planned study using retrospectively obtained tissues. Patients who underwent surgical resection of CRLM at The University of Tokyo Hospital during February 2014 and October 2017 were potential candidates for this study. The study comprises two mass spectrometry experiments using resected specimens. First, we investigated the diagnostic accuracy of PESI-MS and machine learning for CRLM. Next, we used LC-ESI-MS to examine the key molecules that distinguish cancer from non-cancer. This study was approved by the Institutional Ethics Committee of The University of Tokyo, and written informed consent was obtained from all participants.

Of all candidates, patients whose maximum tumor diameter exceeded 5 × 5 × 5 mm were included in the further analysis. We obtained a block of approximately 5 × 5 × 5 mm from surgically resected CRLM and an equivalent block of non-cancer liver parenchyma. If multiple CRLMs were resected, the largest nodule was chosen. If the remaining specimen size following this sample procedure precluded accurate histological analysis, the patients were excluded from this study. Patients with specimens that showed gross necrosis were also excluded. Analysis of liver parenchyma was waived in patients with impaired liver function––Child–Pugh class B and indocyanine green retention rate at 15 min (ICGR15) of > 20.0%––and only the tumor specimen was analyzed. Obtained specimens were immediately frozen in liquid nitrogen and stored at − 80 °C until analysis. LC-ESI-MS was applied for patients in whom both the tumor and the liver parenchyma were analyzed using PESI-MS.

The assessment of the diagnostic accuracy of the logistic regression-based diagnostic algorithm consisted of two steps. First, the mass spectra of each cancerous or non-cancerous tissue were acquired by PESI-MS. Second, all mass spectra were learned by logistic regression to discriminate the blind samples. Furthermore, the discriminant accuracy of diagnostic algorithm was validated using 20 independent specimens that were obtained during surgery undertaken in 2018.

### PESI-MS and machine learning

PESI-MS measurements are described in more detail in a previous report [13]. Briefly, for sample preparation before PESI-MS, 2.5 mg of tissue was homogenized in 100 μl of 50% ethanol using a disposal pestle (Argos Technologies, Vernon Hills, IL, USA). The homogenate was centrifuged at 15,000×*g* for 5 min, and the supernatant was diluted by 50% ethanol to 4-fold for positive ion mode and 2-fold for negative ion mode. Nine microliters of sample solution were placed in the sample plate (Shimadzu Corp., Kyoto, Japan) to perform PESI-MS.

**Fig. 1:**
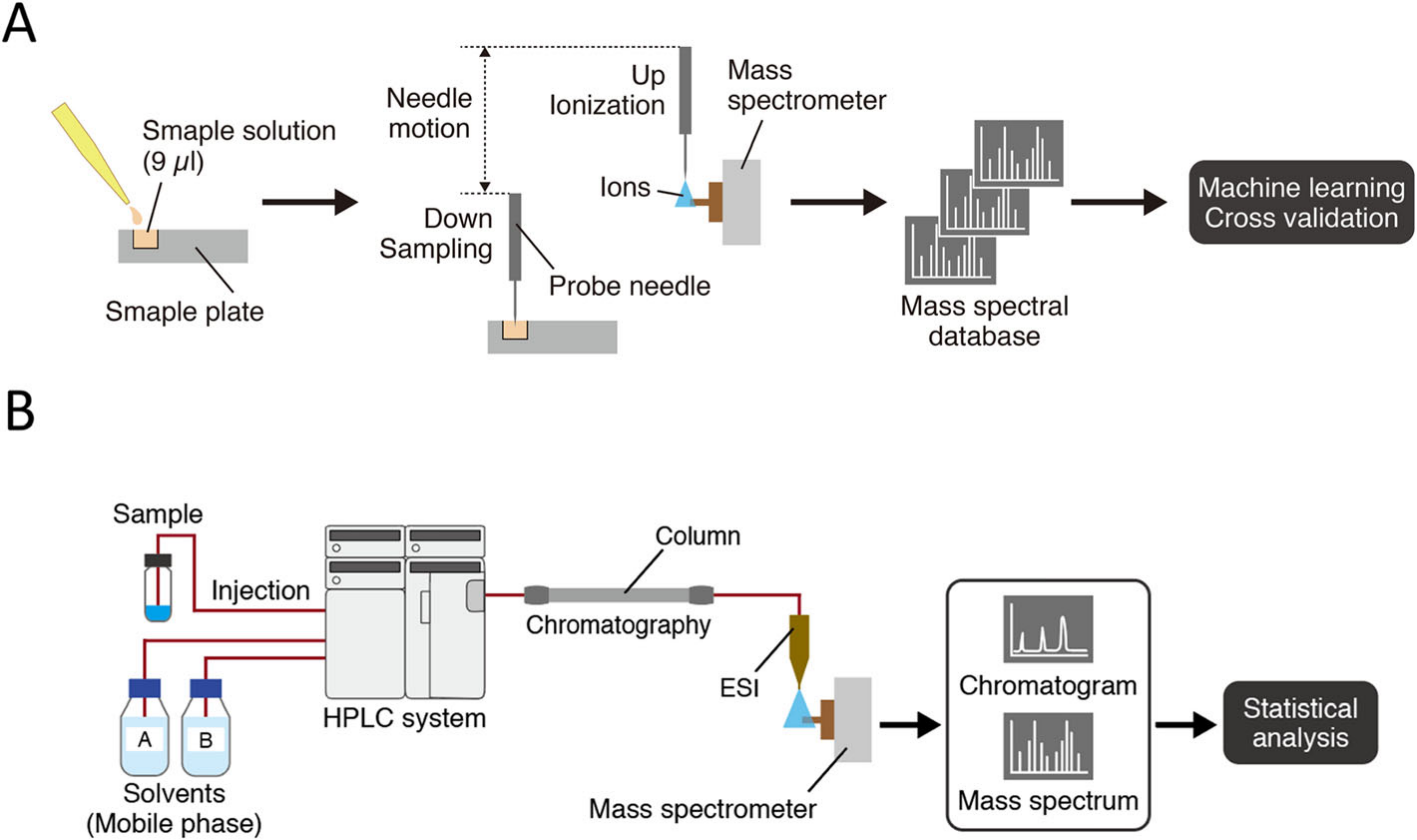
Workflow of **a** probe electrospray ionization-mass spectrometry (PESI-MS) and machine learning and **b** liquid chromatography-electrospray ionization-mass spectrometry (LC-ESI-MS). In PESI-MS, a small amount of sample solution (9 μL) is placed onto the sample plate and directly analyzed without any further pretreatments. In this system, we use all spectral peaks to construct the database instead of annotating each spectral peak. In contrast, we separate the analytes by liquid chromatography in LC-ESI-MS, followed by annotation of each molecule. The data are further evaluated by statistical methods

Ambient ionization unit (DPiMS-8060; Shimadzu Corp.) was used for PESI combined with a triple quadrupole mass spectrometer (LCMS-8060; Shimadzu Corp.) for direct MS, and the analyses were performed as previously described [12]. Analyses were performed for both positive and negative ion mode. The probe needle with a tip radius of < 1 μm was moved downward to touch the sample solution and then upward to apply high voltage (2.3 kV for positive and − 2.0 kV for negative ion mode) for ESI. This movement was repeated, and generated ions were introduced into the mass spectrometer. Figure 1a shows the procedure of PESI-MS analysis. Acquisition of scanning data was completed within approximately 10 min after beginning the sample preparation. Representative mass spectra of each sample were generated using LabSolutions (Shimadzu Corp.). The abscissa indicates mass-to-charge ratio (*m/z*), and the ordinate shows ion intensity (label-free quantification).

Logistic regression analysis was applied to each mass spectrum pattern and corresponding tissue type, that is, CRLM or non-cancerous liver. The expression levels of analyzed lipids obtained from each sample were individually normalized by the median value. Normalized datasets of CRLM and normal liver parenchyma were learned by logistic regression, a type of machine learning, and blinded samples were classified as cancer or not. The possibility of cancer was indicated as the value of probability (0.0–1.0). The detailed procedure, mathematical formula of data processing and discriminant analysis were previously described [14]. To evaluate the discriminative accuracy of the algorithm, leave-one-out cross validation (LOOCV) and 10-fold cross validation (10-fold CV) were applied. LOOCV procedure is summarized as follows. One sample is left out of all samples; this one is considered as the validation set and the remaining samples are assumed as the training set. This cycle is repeated until all samples enter the test set [15]. The 10-fold CV procedure was as follows. Original samples were randomly separated into 10 equally sized subgroups. Of the 10 subgroups, a single subgroup was retained as the blind data for testing the model, and the remaining nine subgroups were used as training data. The procedure was repeated for all subgroups, with each of the 10 subgroups used exactly once as the blind data. The 10 results were then averaged (or otherwise combined) to produce a single estimation. The 10-fold CV for randomly constructed subgroups was performed 10 times. Additionally, the discriminant accuracy of the diagnostic algorithm was further validated using specimens from additional patients with 40 data sets (20 CRLMs and 20 non-cancerous tissue).

### LC-ESI-MS

For LC-ESI-MS, 1 mg of tissue was homogenized in 100 μl of 0.1% formic acid in methanol using a disposal pestle as described above, and the homogenate was mixed using a ThermoMixer C (Eppendorf, Hamburg, Germany) for 5 min at 4 °C. After standing on ice for 5 min, the homogenate was centrifuged at 15,000×*g* for 5 min. The resulting supernatant was diluted by methanol to 50-fold, and 500 μl of sample solution was placed in a LabTotal Vial (Shimadzu Corp.) to perform LC-ESI-MS.

In LC-ESI-MS, high-pressure liquid chromatography (Nexera X2; Shimadzu Corp.) and ESI unit were installed to LCMS-8060 (Fig. 1b). For analysis of tissue components, LC/MS/MS Method Package for Phospholipid Profiling (Shimadzu Corp.) was applied in accordance with the manufacturer’s instructions. A Kinetex C8 column (Kinetex C8, 150 mm × 2.1 mm i.d., 3.6-μm particle size; Phenomenex, Torrance, CA, USA), mobile phase A (20 mM ammonium formate in water) and mobile phase B (acetonitrile: isopropanol 1:1 v/v) were used for LC separation. The concentration of mobile phase B was programmed as 20% (0 min) – 20.0% (1 min) – 40.0% (2 min) – 92.5% (25 min). The oven temperature was 45 °C. Data processing and molecular identification/quantification were performed automatically using LabSolutions software (ver. 5.82 SP1; Shimadzu Corp., Kyoto, Japan).

PESI-MS presents mass spectra associated with molecules of up to 2000 m/z, which include most phospholipids. A total of 457 ion intensities of phospholipids with definite numbers of carbon atoms and double-bonded acyl groups were analyzed.

### Statistical analysis

Continuous variables are expressed as median and range. ROC curves were drawn using probability data from logistic regression (threshold value: 0.500). The absolute ion intensities obtained from LC-ESI-MS were compared using Mann–Whitney *U* test. Statistical analyses were performed using SPSS Statistics, version 25.0 (IBM Corp., Armonk, NY, USA).

## Results

### Patient characteristics

**Fig. 2:**
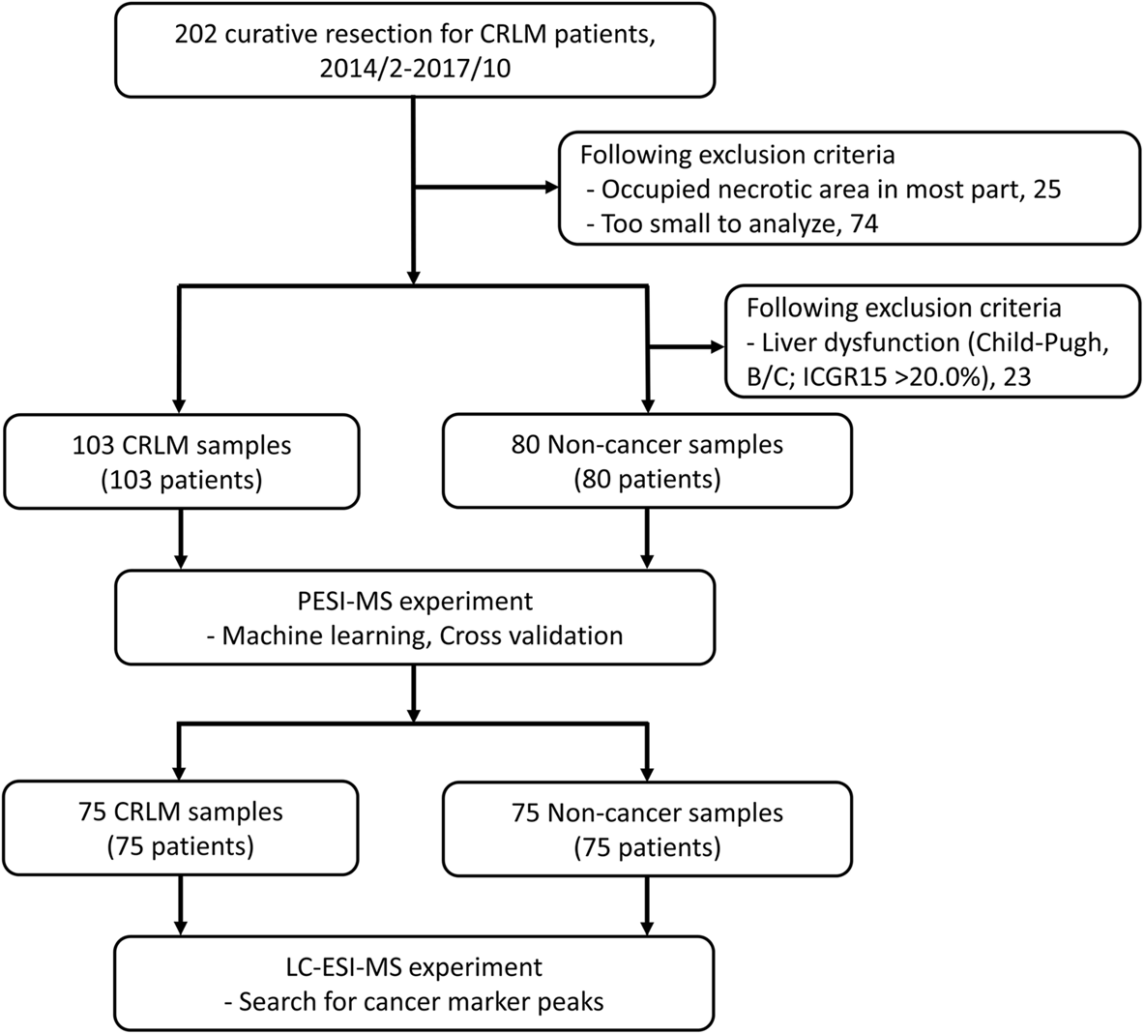
Diagram showing the registry process of patients in this study. All 183 specimens composed of both cancer and non-cancer tissues were used in the PESI-MS experiment, and 150 were used in the LC-ESI-MS experiment

A flow chart of patient selection and sample selection is shown in Fig. 2. Among 202 patients who underwent surgical resection of CRLMs, 99 were excluded because they met either of the following criteria: (1) a tumor smaller than 5 × 5 × 5 mm or a small tumor volume that did not allow qualitative histological examination after sampling for MS (*n* = 74), or (2) a necrotic area that occupied most of the tumor (*n* = 25). Therefore, we made 103 CRLM and non-cancer background liver specimens as candidates of this study. Of these 103 non-cancer liver specimens, 23 were excluded from the analysis because they were obtained from patients with impaired liver function (Child–Pugh class B or ICGR15 of > 20.0%). Finally, PESI-MS was performed on 103 CRLM specimens and 80 non-cancer liver specimens. Next, the matched CRLM and non-cancerous samples from each patient were selected to apply LC-ESI-MS. Hence, 75 CRLM and normal liver parenchyma could be re-analyzed. The demographics and clinicopathological features of the 103 patients with CRLM are summarized in Table 1. Forty-four patients (43%) underwent neoadjuvant chemotherapy.

**Table 1:** Clinicopathological background of patients with CRLM

Variables	*n* = 103
Age, year	68 (35–84)
Sex, M/F*	61 (59.2) / 42 (40.8)
Primary, Colon/Rectum	65 (63.1) / 38 (36.9)
Timing, synchronous/metachronous	64 (62.1) / 39 (37.9)
Child-Pugh classification, A/B/C	95 (92.2) / 8 (7.8) / 0 (0.0)
ICGR15, %	8.7 (0.1–24.5)
CEA, ng/ml	9.4 (0.6–625.5)
CA19–9, IU/ml	21 (1–1400)
Neoadjuvant chemotherapy	44 (42.7)
Adjuvant chemotherapy	40 (38.8)
Number of nodules	3 (1–20)
Maximum tumor diameter, cm	2.4 (0.5–15.0)
KRAS, wild type	54 (56.8)
Differentiation, well/mod/por	21 (26.6) / 53 (67.1) / 5 (6.3)

Values are described as median (range) or n (%)

*M* male, *F* female, *CRLM* colorectal liver metastasis, *ICGR15* indocyanine green retention rate at 15 min, *CEA* carcinoembryonic antigen, *CA19–9* carbohydrate antigen 19–9

### PESI-MS and machine learning

**Fig. 3:**
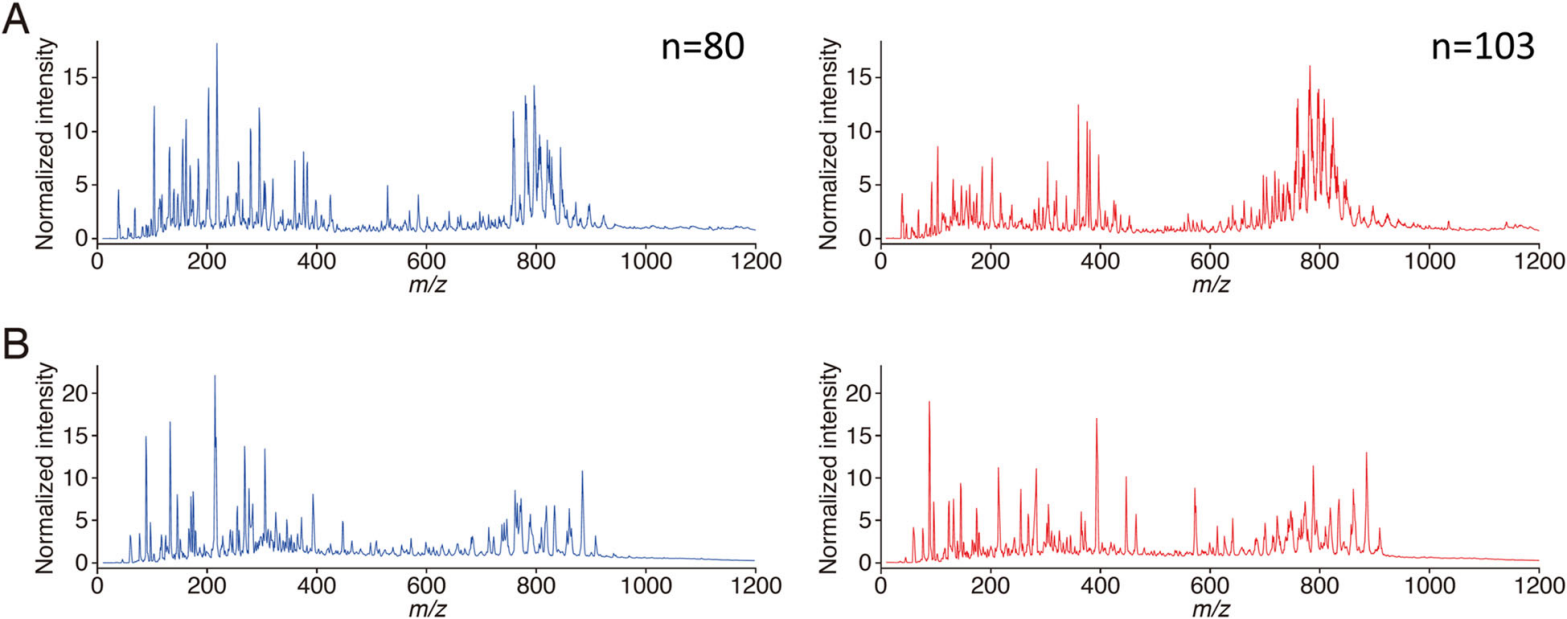
Normalized mean mass spectra by PESI-MS. Both **a** positive and **b** negative ion modes are shown. Note that overt differences in ion species were not apparent between the positive and negative ion modes. Blue: non-cancer liver parenchyma. Red: colorectal liver metastasis

**Fig. 4:**
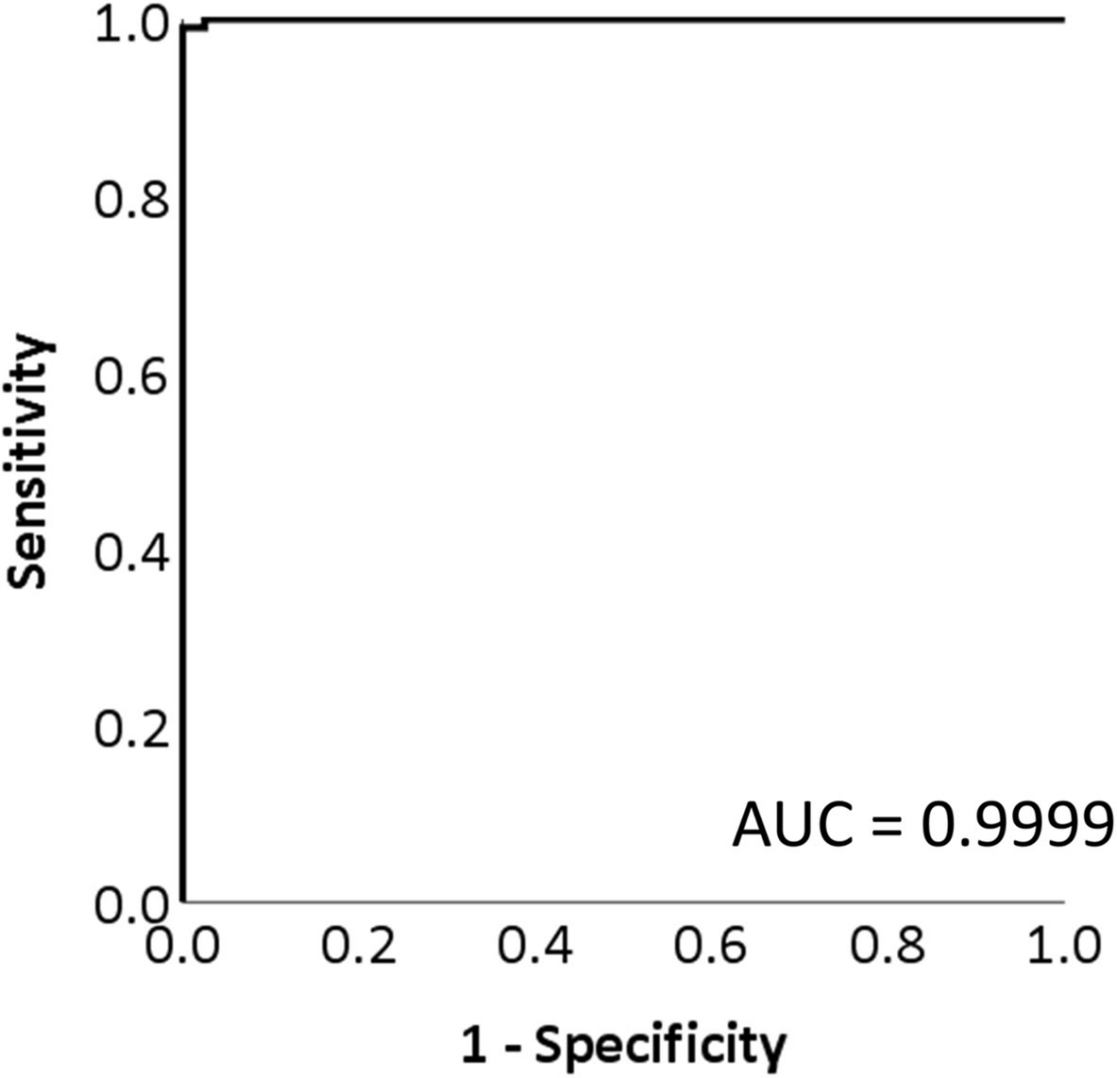
Receiver operating characteristic curve of probability by discriminant analysis. By discriminating the spectral data by machine learning, the area under the curve (AUC) was 0.9999; this value achieved both extraordinarily high sensitivity and specificity

Normalized mean mass spectra of CRLM and non-cancer liver parenchymal tissue are shown in Fig. 3. The mass spectral pattern of each category differed in positive and negative ion modes. These spectral patterns were learned and analyzed using logistic regression and validated using LOOCV method. Among 103 CRLM samples, 102 samples were correctly diagnosed. Meanwhile, all 80 non-cancer samples were correctly diagnosed. Specificity, sensitivity and accuracy of logistic regression were 100, 99 and 99%, respectively. Ten-fold CV demonstrated that the total accuracy rate was 99.0%. (Supplementary Table 1). ROC curve of logistic regression for discriminating CRLM is shown in Fig. 4, in which the area under the curve was 0.9999.

In the independent validation set, 18 CRLMs were correctly diagnosed, while all 20 non-cancerous samples were correctly diagnosed. Specificity and sensitivity were 100.0 and 90.0% respectively (Supplementary Table 2).

### LC-ESI-MS

**Fig. 5:**
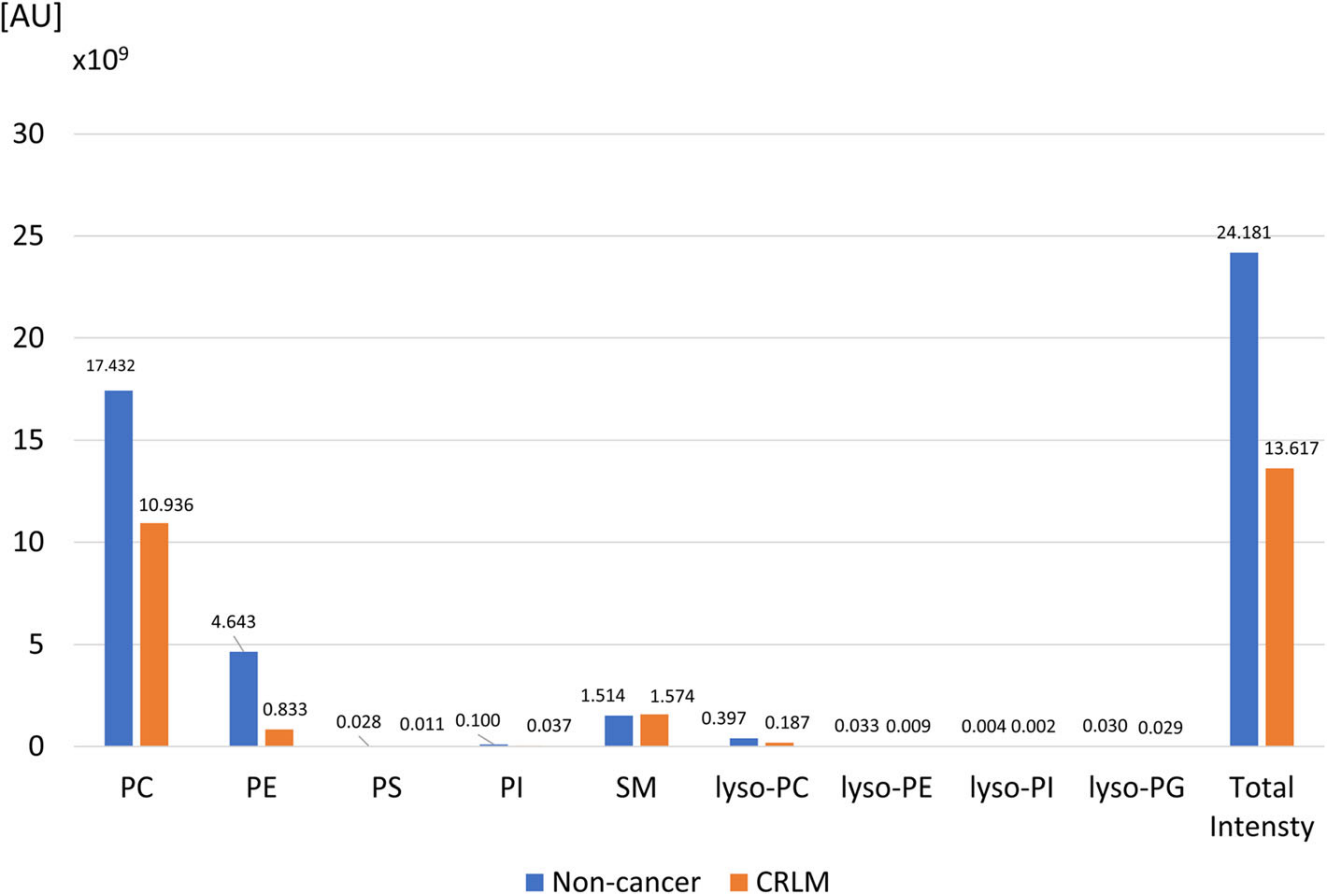
Comparison of lipid contents between non-cancer liver parenchyma and colorectal liver metastasis. Note that the amount of both phosphatidylcholine and phosphatidylethanolamine were significantly lower in colorectal liver metastasis (*P* < 0.01, respectively). PC, phosphatidylcholine; PE, phosphatidylethanolamine; PS, phosphatidylserine; PI, phosphatidylinositol; SM, sphingomyelin

We identified 457 species of phospholipids, among which 146 ion (31.9%) species showed significantly different ion intensities between CRLM and non-cancer liver parenchyma (*P* < 0.01). Table 2 shows top 10 phospholipids among these 146 species. The total ion intensity of each phospholipid, including phosphatidylcholine, phosphatidylethanolamine, phosphatidylserine, phosphatidylinositol, sphingomyelin, lysophosphatidylcholine, lysophosphatidylethanolamine, lysophosphatidylinositol and lysophosphatidylglycerol, was compared between CRLM and non-cancer liver parenchyma (Fig. 5). Notably, the expression of phosphatidylcholine and phosphatidylethanolamine in the non-cancer liver parenchyma (mean: 2.32 × 10^8^ arbitrary unit [AU] and 6.19 × 10^7^ AU, respectively) was higher than CRLM (mean: 1.46 × 10^8^ AU and 1.11 × 10^7^ AU; *P* < 0.01, respectively).

**Table 2:** Candidate marker of phospholipids to discriminate CRLM

m/z	Candidate molecule	Median ion intensity		*P* value (−log10)
		Non-cancer	CRLM	
*Dominant in non-cancer liver specimen*				
760.50	PE (38:7–16:1/22:6)	12,970.0	0.0	28.3
712.50	PE (34:3–16:0/18:3)	19,792.0	0.0	27.7
788.55	PE (40:7–18:1/22:6)	153,466.0	0.0	26.8
806.50	PS (40:7–18:1/22:6)	8562.0	0.0	27.0
863.55	PI (36:1–16:0/20:1)	8974.0	0.0	25.6
762.50	PE (38:6–16:0/22:6)	2,058,266.0	57,728.0	25.5
736.50	PE (36:5–16:0/20:5)	121,756.0	11,973.0	25.4
850.55	PC (38:6–16:0/22:6)	480,734.0	29,393.0	25.4
738.50	PE (36:4–16:0/20:4)	566,367.0	63,337.0	25.4
790.55	PE (40:6–18:0/22:6)	1,171,554.0	66,685.0	25.4
*Dominant in CRLM specimen*				
747.55	SM (34:1-d18:1/16:0)	175,390.0	375,704.0	20.2
830.60	PC (36:2–16:0/20:2)	0.0	9619.0	19.9
800.60	PE (40:1–18:1/22:0)	0.0	17,383.0	18.6
856.60	PC (38:3–18:1/20:2)	0.0	8239.0	18.2
768.55	PE (38:3–18:1/20:2)	0.0	39,983.0	16.3
858.60	PC (38:2–18:1/20:1)	0.0	18,930.0	16.3
766.55	PE (38:4–18:2/20:2)	0.0	17,581.0	14.1
830.60	PC (36:2–16:1/20:1)	0.0	8761.0	10.9
716.55	PE (34:1–16:1/18:0)	0.0	65,533.0	10.9
786.55	PS (36:2–18:1/18:1)	0.0	3703.0	8.4

*CRLM* colorectal liver metastasis, *PE* phosphatidylethanolamine, *PC* phosphatidylcholine, *SM* sphingomyelin, *LPC* lysophosphatidylcholine

As shown in Table 2, phospholipid species that are upregulated in non-cancer liver tissues are more likely to contain fatty acids (no double bonds exist in the carbon chain) and polyunsaturated fatty acids (more than two double bonds exist in the carbon chain), while those increased in CRLM are predominantly monounsaturated fatty acids (one double bond exists in the carbon chain). The total ion intensities of saturated, monounsaturated, and polyunsaturated fatty acids were calculated in CRLM tissue and non-cancer liver parenchyma. Monounsaturated fatty acids accounted for 37% of total phospholipids in CRLM and 11% of those in non-cancer liver specimens (*P* < 0.01). Conversely, the total ion intensity of the other two saturated fatty acids was higher in non-cancer liver tissue than in CRLM (Table 3).

**Table 3:** Degree of FA saturation

	Saturated FAs	Mono-unsaturated FAs	Poly-unsaturated FAs
Non-cancer	46.8%	10.7%	42.5%
CRLM	39.2%	37.2%	23.6%

*FA* fatty acids, *CRLM* colorectal liver metastasis

## Discussion

This new rapid diagnostic system showed high accuracy rate (99%) in discriminating CRLM tumor from non-cancer liver parenchyma. LC-ESI-MS analysis revealed that phospholipids categorized as monounsaturated fatty acids were more highly expressed in CRLM than in non-cancer liver parenchyma. The most distinctive feature of PESI-MS is that it provides mass spectra of individual tissue rapidly using small amounts of sample. These characteristics are advantageous in clinical situations. Crucially, the precise differential diagnosis of new hepatic lesions found intraoperatively can be completed during the operation. This new diagnostic system has the potential to supersede the mainstream––but less accurate––methods of intraoperative biopsy and frozen section diagnosis of liver lesions that are unexpectedly found during surgery [9].

In late years, other types of ambient mass spectrometry, including intelligent knife, which is composed of rapid evaporative ionization mass spectrometry, or MasSpec Pen, which is composed of desorption electrospray ionization mass spectrometry have been studied in clinical settings [16, 17]. These techniques are classified in the same category as PESI-MS, and do not require chromatographic separation. PESI is a new ionization method that does not require the desalting procedure and can ionize a large variety of molecular components that are difficult to ionize by the former two modalities. Additionally, it is easier to manipulate PESI-MS compared with conventional mass-spectrometry. An operator only has to push the “acquire” button following the setting of the sample plate on the machine interface, while LC-MS requires dedicated column, chromatographic and MS/MS conditions. Although the initial cost is nearly equal to other mass spectrometer, it is maintenance-free for PESI.

This new diagnostic system could be effective in intraoperative evaluation of surgical margin, lymph node metastasis and disappearing CRLM after chemotherapy. CRLM frequently invades intrahepatic vascular structures, which could affect the postoperative prognosis [4, 18]. However, a surgical margin is sometimes judged negative at intraoperative examination using frozen section although postoperative examination using paraffin-embedded sections did positive [19]. Similarly, a small foci of cancerous cells in a resected lymph node is sometimes confirmed only after operation. These diagnostic discrepancies would be attributable to the small sample volume. PESI-MS and machine learning can detect cancerous tissue as small as 2.5 mg, which might help to overcome this difficulty in diagnosing surgical margin and lymph node metastasis. Resection of disappearing CRLM after effective chemotherapy remains a clinical challenge because such a tumor is difficult to identify during operation. In such cases, surgeons search for tumor in the resected specimen. However, any suspicious lesion in the specimen slices is sometimes missed because of the small tumor size. PESI-MS might be sensitive in detecting tumor cells in such specimen even if tumor is not visible. Further studies are required to analyze the surgical margin, lymph node and disappearing CRLM using this new diagnostic system.

Approximately one-third of the 465 investigated phospholipids showed significantly different ion intensity levels between CRLM and non-cancer liver parenchyma by LC-ESI-MS analysis. This was in good agreement with previous reports demonstrating significant changes in lipid profiles in cancer patients [20–22]. Other reports have demonstrated the high discriminant accuracy of PESI-MS and machine learning for head and neck cancer and breast cancer (92.9 and 96.0%, respectively) [12, 23]. Similarly, this system also could rapidly discriminate CRLM from non-cancerous tissue. In particular, our study confirmed the results of a previous study showing that phosphatidylcholine and phosphatidylethanolamine are highly expressed in normal hepatocyte (*P* < 0.01; Fig. 5) [24].

Monounsaturated fatty acids were recently reported to be correlated with tumor biology [25–27]. In the present study, monounsaturated fatty acids comprised 37.2% of all investigated phospholipids, and the proportion was much higher than that in non-cancer liver parenchyma (10.7%, P < 0.01). This is consistent with previous reports demonstrating that the expression level of phospholipids with monounsaturated fatty acids was higher in colorectal cancer than in normal mucosa (Table 4) [28–30]. There are no reports referring to unsaturation of fatty acids in CRLM. Our findings support the application of cancer therapy that targets the enzymes expressed in the endoplasmic reticulum, such as SCD-1, to CRLM [27, 31]. However, further experiments are required to identify the exact molecules comprising monounsaturated fatty acids in CRLM.

**Table 4:** Previously reported lipid biomarkers in colorectal liver metastasis

Author	Year	Sample size	Modality	Name of lipid	Sample type	Up/Down	Remarks
Shimma [28]	2007	1	MALDI-MS	SM (16:0)	Tissue	Up	Diagnostic Factor
Thomas [29]	2013	3	MALDI-MS	PE (38:6), PE (40:4)	Tissue	Up	Diagnostic Factor
Figueiredo [30]	2018	36	MALDI-MS	Sphingolipids	Tissue	Up	Diagnostic Factor
				Glycerophospholipid			Prognostic Factor
Present	2020	75	LC-ESI-MS	Mono-unsaturated fatty acid	Tissue	Up	Diagnostic Factor

*MALDI-MS* matrix-assisted laser desorption/ionization-mass spectrometry, *LC-ESI-MS* liquid chromatography-electrospray ionization-mass spectrometry, *SM* sphingomyelin, *PE* phosphatidylethanolamine

This study has several potential limitations. First, the present study was retrospective and the sample size was small. The new diagnostic system was applied after making final histological diagnosis. Additionally, we excluded a number of samples because they were prepared only from patients whose tumor diameter was large enough after excision for indisputable pathological evaluation. A further prospective study may be warranted. Second, some patients underwent neoadjuvant chemotherapy, which may lead to changes in the lipid profiles of metastatic lesions. Incorrect diagnosis in PESI-MS and machine learning was seen in only one specimen, which was obtained from non-cancerous liver in the patient who underwent 59 sessions of preoperative chemotherapy (FOLFOX or FOLFIRI with bevacizumab). Further studies focusing on the use of this new diagnostic system in the patients undergoing neoadjuvant chemotherapy may be favored.

## Conclusions

The new combination of PESI-MS and machine learning is likely to provide a higher degree of precision in discriminating CRLM from non-cancerous liver tissue. Additional analysis using LC-ESI-MS is required to identify specific monounsaturated fatty acid-bonded phospholipids as CRLM biomarker candidates.

## Supplementary Information

**Additional file 1: Supplementary Table 1.** Discriminant accuracy of 10 times 10-fold cross validation for 103 CRLM and 80 non-cancerous liver parenchyma.

**Additional file 2: Supplementary Table 2.** Discriminant accuracy of algorithm for independent validation dataset obtained from 20 CRLM and 20 non-cancerous liver parenchyma.

## Data Availability

The datasets used and /or analyzed during the current study are available from the corresponding author on reasonable request.

## Abbreviations

CRLM: Colorectal liver metastasis
PESI-MS: Probe electrospray ionization-mass spectrometry
LC-ESI-MS: Liquid chromatography-electrospray ionization-mass spectrometry
ICGR15: Indocyanine green retention rate at 15 min
LOOCV: Leave-one-out cross validation
