# Supplementary material for: A new rapid diagnostic system with ambient mass spectrometry and machine learning for colorectal liver metastasis
Source: BMC Cancer. 2021 Mar 10;21:262. doi: 10.1186/s12885-021-08001-5 (PMC7945316; doi:10.1186/s12885-021-08001-5)
Supplement: Supplementary file 1 — Additional file 1: Supplementary Table 1. Discriminant accuracy of 10 times 10-fold cross validation for 103 CRLM and 80 non-cancerous liver parenchyma. [file 12885_2021_8001_MOESM1_ESM.docx]

**Supplementary Table 1. Discriminant accuracy of 10 times 10-fold cross validation for 103 CRLM and 80 non-cancerous liver parenchyma.**

| Number of subgroups | 1 | 2 | 3 | 4 | 5 | 6 | 7 | 8 | 9 | 10 |
| --- | --- | --- | --- | --- | --- | --- | --- | --- | --- | --- |
| 1 | 100.0% | 100.0% | 100.0% | 100.0% | 100.0% | 100.0% | 100.0% | 100.0% | 100.0% | 94.7% |
| 2 | 100.0% | 100.0% | 100.0% | 100.0% | 100.0% | 100.0% | 100.0% | 100.0% | 100.0% | 100.0% |
| 3 | 100.0% | 100.0% | 100.0% | 94.7% | 100.0% | 100.0% | 100.0% | 100.0% | 100.0% | 100.0% |
| 4 | 100.0% | 94.4% | 100.0% | 100.0% | 100.0% | 94.4% | 100.0% | 100.0% | 94.4% | 100.0% |
| 5 | 94.4% | 100.0% | 100.0% | 94.4% | 100.0% | 100.0% | 100.0% | 94.4% | 100.0% | 94.4% |
| 6 | 94.4% | 100.0% | 94.4% | 100.0% | 100.0% | 100.0% | 100.0% | 100.0% | 94.4% | 100.0% |
| 7 | 100.0% | 100.0% | 100.0% | 100.0% | 94.4% | 100.0% | 100.0% | 100.0% | 100.0% | 100.0% |
| 8 | 100.0% | 100.0% | 100.0% | 100.0% | 94.4% | 100.0% | 100.0% | 100.0% | 100.0% | 100.0% |
| 9 | 94.4% | 100.0% | 100.0% | 100.0% | 100.0% | 94.4% | 94.4% | 100.0% | 100.0% | 100.0% |
| 10 | 100.0% | 100.0% | 94.4% | 100.0% | 100.0% | 100.0% | 100.0% | 100.0% | 100.0% | 100.0% |
| Mean | 98.9% | 99.5% | 98.9% | 99.5% | 98.9% | 98.9% | 99.5% | 99.5% | 98.9% | 98.9% |

CRLM, colorectal liver metastasis
