# Supplementary material for: A new rapid diagnostic system with ambient mass spectrometry and machine learning for colorectal liver metastasis
Source: BMC Cancer. 2021 Mar 10;21:262. doi: 10.1186/s12885-021-08001-5 (PMC7945316; doi:10.1186/s12885-021-08001-5)
Supplement: Supplementary file 2 — Additional file 2: Supplementary Table 2. Discriminant accuracy of algorithm for independent validation dataset obtained from 20 CRLM and 20 non-cancerous liver parenchyma. [file 12885_2021_8001_MOESM2_ESM.docx]

**Supplementary Table 2. Discriminant accuracy of algorithm for independent validation dataset obtained from 20 CRLM and 20 non-cancerous liver parenchyma.**

| CRLM or Non-cancer | n | Correct | Failure | % |  |
| --- | --- | --- | --- | --- | --- |
| Non-cancer | 20 | 20 | 0 | 100.0 | (=specificity) |
| CRLM | 20 | 18 | 2 | 90.0 | (=sensitivity) |
| Total | 40 | 38 | 2 | 95.0 | (=accuracy rate) |

CRLM, colorectal liver metastasis
